# Psychogenic Stress in Hospitalized Dogs: Cross Species Comparisons, Implications for Health Care, and the Challenges of Evaluation

**DOI:** 10.3390/ani4020331

**Published:** 2014-06-16

**Authors:** Jessica P. Hekman, Alicia Z. Karas, Claire R. Sharp

**Affiliations:** 1 Department of Animal Sciences, University of Illinois at Urbana-Champaign, 1207 West Gregory Drive, Urbana, IL 61801, USA; E-Mail: hekman2@illinois.edu; 2 Department of Clinical Sciences, Cummings School of Veterinary Medicine, Tufts University, 200 Westboro Road, North Grafton, MA 01536, USA; E-Mail: claire.sharp@tufts.edu

**Keywords:** stress, hospitalization, dogs

## Abstract

**Simple Summary:**

The effects of stress on health outcomes in animals are well documented. Veterinary clinicians may be able to improve their patients’ care by better understanding how to recognize and reduce stress in those patients. This review will describe the physiology of the mammalian stress response and known health consequences of psychogenic, rather than physical, stress; as well as methods of measuring stress in animals. While the review will address stress in a range of domestic species, it will specifically focus on dogs.

**Abstract:**

Evidence to support the existence of health consequences of psychogenic stress has been documented across a range of domestic species. A general understanding of methods of recognition and means of mitigation of psychogenic stress in hospitalized animals is arguably an important feature of the continuing efforts of clinicians to improve the well-being and health of dogs and other veterinary patients. The intent of this review is to describe, in a variety of species: the physiology of the stress syndrome, with particular attention to the hypothalamic-pituitary-adrenal axis; causes and characteristics of psychogenic stress; mechanisms and sequelae of stress-induced immune dysfunction; and other adverse effects of stress on health outcomes. Following that, we describe general aspects of the measurement of stress and the role of physiological measures and behavioral signals that may predict stress in hospitalized animals, specifically focusing on dogs.

## 1. Introduction

In recent decades, the effects of stress on human health, and methods to reduce its prevalence, have received a great deal of attention. It is a long recognized phenomenon that husbandry and transport stress can dramatically affect animal health in livestock, and stress is beginning to be recognized as a factor in naturally occurring disease in humans [1,2]. The clinical relevance of stress in small animal veterinary patients has not been examined, but it seems likely that the impact of stress on clinical outcomes, such as survival rates or the speed of recovery from surgery, is under-appreciated. The implications that stress has for human health care outcomes and health in livestock serve as a foundation from which to extrapolate an analogous concern for our small animal veterinary patients. Reducing stress in companion animals under veterinary care is clearly important for their mental well-being, and is even more important if pharmacologic or non-pharmacologic interventions can also prevent disease or improve healthcare outcomes.

The experience of hospitalization may be expected to include several factors which are known to induce stress in veterinary species such as the dog, including separation from the primary caretaker [3], environment [4], novel stimuli [5], increased noise levels [6], and a constrained environment [7,8]. An investigation of behavior and heart rate of pre-operative hospitalized dogs suggests that these animals experience stress [9], yet there is little detail regarding the effects of such stress on health outcomes for dogs. This is likely in part due to a lack of understanding of stress by veterinary clinicians, as well as a lack of tools for accurate identification and quantification of stress in dogs. Investigation of the causes and effects of stress on the hospitalized small animal patient requires an understanding of both its physiology and pathophysiology, and of available methods of quantification in that particular species. The purpose of this review is to describe the physiology of the stress syndrome, characteristics of stressors, stress-induced immune dysfunction, and other adverse effects of stress on health using a cross-species approach. Following that, general aspects of the measurement of stress and the role of behavioral signals that may predict it are described, specifically focusing on dogs.

## 2. Physiology of the Stress Syndrome

The stress response is a normal part of daily life, and is only harmful when triggered too intensely or for too long [10,11]. Peripheral expression of the stress response is modulated via two systems, the sympatho-adreno-medullary (SAM) axis and the hypothalamic-pituitary-adrenal (HPA) axis.

The SAM axis mediates the well-known “fight or flight” response, an initial, rapid response to an immediate stressor. Activation of the sympathetic nervous system and subsequent release of catecholamines (epinephrine and norephinephrine) from sympathetic nerve terminals and the adrenal medulla results in a state of physiologic readiness for response. Manifestations of SAM axis activation include mydriasis, increased heart rate, increased blood pressure, cutaneous vasoconstriction, an alert state, and increased plasma glucose and free fatty acid concentrations [12].

A slower response to a stressor, with effects in minutes to hours or days, is mediated by activation of the HPA axis leading to the release of glucocorticoids (GCs) from the adrenal cortex. This endocrine portion of the mammalian stress response originates in the hypothalamus, with release of corticotropin releasing hormone and arginine vasopressin. These hormones in turn stimulate the release of adrenocorticotropic hormone from the pituitary gland, resulting in the production and release of GCs from the adrenal glands. Peripherally circulating GCs, cortisol and corticosterone, provide negative feedback to this system [12]. Glucocorticoids influence a large number of metabolic processes, including protein, glucose, and fatty acid metabolism, and immune function [13], and can induce a catabolic state [14], while corticotropin releasing hormone suppreses gastro-intestinal motility [15,16] and arginine vasopressin regulates the glomerular filtration rate (GFR), cAMP generation, and fluid balance [17]. Acting jointly, these hormones can also influence growth, thyroid function, and reproduction [18].

## 3. Classification and Causes of Stress

Stress can be classified in a variety of ways, and different classifications of stress may have different consequences for health outcomes. Stress can be acute or chronic. In the laboratory setting, acute stress is sometimes defined as having a duration of less than one hour [19]. Chronic stressors in the laboratory often persist for 4–5 days, though chronic psychosocial stress in humans can last years [20]. These definitions differ from clinical perceptions of acute *versus* chronic disease, in which a disease lasting as long as several weeks may still be considered acute [21,22].

The term “stress” covers several different concepts: physiologic stress, non-physiologic or psychogenic stress, and distress [10]. Physiologic stress describes exposure to positive or negative physical, systemic, or environmental challenges that perturb the body’s homeostasis. In a veterinary setting, negative physiologic stress may be induced by systemic illness, trauma, and surgery. Similarly, psychogenic stress describes exposure to psychological or social challenges which result in disruption of psychological well-being. Negative psychogenic stress in a domesticated animal may be induced by separation from a caretaker, being subjected to invasive procedures in the absence of familiar caretakers, or exposure to a novel environment [23]. Positive psychogenic stress has been less widely studied, but may be understood to refer to situations such as reunion with a caretaker or engagement in a highly anticipated game such as fetch. Negative psychogenic stressors may account for some degree of avoidable morbidity in medical care, if measures to reduce stressors exist. Both physiologic and psychogenic stress are a normal part of life, and the healthy body and mind can adapt to maintain normal function [11]. Stress becomes distress when the body cannot restore homeostasis in the face of overwhelming physiologic stress, or when overwhelming psychogenic stress threatens mental well-being [10]. When marked, stress is associated with numerous pathophysiological sequellae, ranging from poor mental to poor physical well-being. This review is concerned with negative psychogenic stress, especially as it proceeds to distress. Following the stress literature, the term “stress” will be used to refer to “distress.”

Psychogenic stressors may be classified as social/non-social [24] or controllable/uncontrollable [25]. A social stressor involves aversive interactions with a hostile conspecific, such as aggressive dogs in facing cages, or separation from the attachment figure, as opposed to a nonsocial stressor, such as exposure to aversive environmental conditions, such as elevated noise levels. An uncontrollable stressor is not escapable by the animal, such as inescapable electric shock or restraint for medical procedures, as opposed to a controllable stressor, such as social stress mitigated by retreat to the rear of the cage. All of these types of stressors might be present in a veterinary medical setting; hostile conspecifics might present a social stressor, and restraint for a medical procedure might present an uncontrollable stressor. Practically, social stressors due to a lack of accommodation to canine body language and needs by their human caregiver are also uncontrollable, and are arguably widely encountered in veterinary care.

Different stressors are known to cause varying levels of activation of metabolic and endocrine responses in laboratory animals [26,27], and may also have varying consequences in hospitalized animals. For example, social stressors such as defeat in conflict with a conspecific activate the sympathetic nervous system more strongly than non-social stressors (restraint and shock) in rats [24]. Uncontrollable stressors, e.g., stressors from which the animal cannot escape and which cannot be mitigated, appear to activate the stress response more strongly across species than controllable stressors. This has been shown with escapable *versus* inescapable electric shocks in dogs [25]. Enhanced skill in assessment of and response to body language cues, as well as addressing a hospitalized animal’s environmental needs, may produce an overall reduction in psychogenic stress.

## 4. The Effects of Stress on Health Outcomes

There is ample evidence from laboratory, clinical, and epidemiological trials demonstrating that acute and chronic psychogenic stress can result in negative consequences on both human and non-human animals, contributing to increased patient morbidity or mortality [28]. These health implications include, but are not limited to, susceptibility to infection and sepsis, impaired antibody responses to vaccination, slowed wound healing, and development of gastric ulceration [28,29,30]. Chronic stress from anxiety disorders is associated with shortened lifespan in dogs [31]. Outcomes of particular relevance to hospitalized animals will be discussed under the subheadings below.

### 4.1. Interactions between the Stress Response and the Immune System

There is a complex interplay between the stress response and the immune system, which varies based on the duration (acute or chronic), timing (before or after immune challenge), and type (social or non-social, controllable or uncontrollable) of the stressor. The balance between the immunoenhancing and immunosuppressive effects of stress is complex, and any stressor may theoretically be immunoenhancing or immunosuppressive in a particular individual, based on the aforementioned variables.

The initial stress response to a psychogenic stressor or an injury is often immunoenhancing and pro-inflammatory [32]. Acute stress enhances T lymphocyte responsiveness and appropriate differentiation into T helper 1 (Th1) or T helper 2 (Th2) lymphocytes [19,33]. Increased endogenous GC production associated with acute stress may result in a “stress leukogram,” characterized by a peripheral neutrophilia, lymphopenia, eosinopenia, and monocytosis due to redistribution and increased trafficking of white blood cells [34]. Circulating natural killer cell numbers may also increase, and the levels of both pro- and anti-inflammatory cytokines are altered [35,36]. Taken together, these changes prepare the immune system for an impending challenge, such as microbial invasion.

As time passes and an acute stressor becomes chronic, the effects of the stress response on the immune system may shift from immunoenhancement to immunosuppression. Glucocorticoids can inappropriately suppress the Th1 response and enhance the Th2 response [33]. Chronic stress can also be associated with leukopenia, lymphopenia, and reduced leukocyte phagocytic capacity [37]. These immunosuppressive and anti-inflammatory properties of the chronic stress response may be adaptive to limit systemic inflammation while permitting a controlled, localized inflammatory response to injury or infection, or to shift from the innate immune response to the adaptive immune response when the stressor is of longer duration, allowing enough time for the slower adaptive response to be effective [32,33].

However, when activation of the stress response is exaggerated or prolonged, it may lead to inappropriate immunosuppression [38]. For example, chronic stress induced immunosuppression in humans can lead to increased susceptibility to infection and neoplasia and decreased response to vaccination [28,39]. In mice, chronic restraint and acoustic stress results in decreased ability to eliminate bacterial infection associated with mild peritonitis [38]. 

The timing of the stressor is also important. The immunoenhancing effects of acute stress are best realized when the stressor is timed to occur just before immune challenge. When a stressor occurs after the onset of an immune response, the stressor is more likely to be suppressive, even in the acute setting [19].

In addition to the duration and timing of the stressor, the type of stressor may also affect the pro-*versus* anti-inflammatory consequences of that stress. For example, social disruption stress in mice is pro-inflammatory, and presumed to be an adaptive response, since it is often associated with physical conflict resulting in wounds [40].

Hospitalized animals may be exposed to both acute and chronic stressors of a variety of types, including social, nonsocial, uncontrollable, and controllable. The interactions of immunoenhancing acute stressors *versus* immunosuppressing chronic stressors may be difficult to predict. However, as stressors associated with hospitalization of companion animals often occur after the challenge that initially prompted presentation of the animal to the veterinary hospital, they may be expected to be immunosuppressive, not immunoenhancing. Similarly, as the animal’s time in the hospital extends, the stress of hospitalization becomes more likely to act as chronic rather than acute stress, and therefore becomes more likely to suppress rather than enhance the immune response.

### 4.2. Response to Vaccination

Acute stress, on the order of minutes or hours, is immunoenhancing, so that stress may essentially serve as a natural adjuvant when it occurs at the time of vaccination [19] and has been shown in mice to induce a long-lasting increase in immunity [41] through enhancement of both the adaptive and innate arms of the immune system [42]. Timing is essential, however, because psychogenic stressors occurring after vaccination have been associated with a poorer antibody response [43], and a stressor which becomes chronic may suppress rather than enhance immune activity [19]. These beneficial effects appear to be mediated by endogenous glucocorticoids in physiologic, not pharmacologic, concentrations [19].

### 4.3. Susceptibility to Sepsis

Stress may influence an animal’s susceptibility to sepsis. In sepsis, an exaggerated systemic response to infection occurs, which results in increased mortality and may be more damaging than the original infection [40]. Studies in mice suggest that increased GC concentrations due to psychogenic stress might provide protection against sepsis due to the immunosuppressive effects of GCs, or might increase susceptibility to sepsis due to increased GC resistance, depending on the effects of different stressor types [13,44]. It is not known whether stress might increase or decrease susceptibility to sepsis in hospitalized veterinary patients.

### 4.4. Wound Healing

In humans, both acute and chronic psychogenic stress can negatively affect wound healing, even when activated for as short a time as several hours or days, as may be the case in hospitalized animals [45]. Chronic psychogenic stressors shown to affect wound healing in humans include examination stress, care taking for dementia patients, and marital stress [46]. Additionally, greater amounts of stress perceived pre-operatively in humans predict slower wound healing [45]. Therefore, psychogenic stress during hospitalization might be expected to affect wound healing in veterinary patients with traumatic wounds and surgical incisions.

### 4.5. Gastrointestinal System

Stress has been shown in humans and in laboratory animals to induce the development of stomach ulcers, particularly in conjunction with pathogens such as Helicobacter pylori, or non-steroidal anti-inflammatory drugs [47]. Chronic stress is also associated with the exacerbation of inflammatory bowel disease in humans [48], and stressful environment and anxious personality traits are associated with chronic idiopathic large-bowel disease in dogs [49]. Dogs undergoing significant physiological stress in the form of the physical exertion of a sled race are at increased risk of developing gastric lesions [50]. In addition to physiological stress, sled dogs are arguably exposed to psychogenic stress in the form of exposure to unfamiliar environments, interactions with unfamiliar conspecifics, and the race itself. Similarly, hospitalized animals are exposed to physiologic stressors, in the form of illness or trauma, in addition to psychogenic stressors. However, effects of hospitalization stress on gastrointestinal function in dogs have not yet been investigated.

### 4.6. Cardiovascular System

The detrimental effects of psychogenic stress on cardiovascular function in humans and laboratory animals are well recognized. Acute social stress increases T-wave alternans in normal dogs, and has triggered atrial fibrillation in humans [51,52]. Acute psychogenic stress is associated with hypercoagulability and thrombotic tendencies in humans, which is exacerbated in subjects who are also undergoing chronic stress [53,54]. Additionally, chronic psychogenic stress is associated with coronary artery disease and hypertension in humans, and increased risk of cardiovascular disease occurrence, morbidity, and mortality [55,56,57]. It is not known at this time how stress affects the cardiovascular systems of hospitalized animals with or without heart disease.

## 5. Quantification of Stress

Although the effects of stress on health outcomes have been well studied in humans and animal models, little work has been done to evaluate the effects of different stressors on health outcomes in hospitalized animals with naturally occurring disease. It is likely that, as in other species, effects of stress on specific health outcomes in veterinary patients will vary depending on the duration, timing, and type of stressor(s). In order to better understand how stress affects hospitalized veterinary patients, reliable measurement tools are essential. As various markers of stress can be species specific, the following section focuses primarily on the dog.

Quantification of stress is difficult. Both physiological and behavioral measures have been used to quantify psychogenic stress. However, both approaches have drawbacks, and results may be challenging to interpret. Theoretically, psychogenic stress may be objectively evaluated by measuring the circulating concentrations of SAM or HPA axis hormones, or associated physiologic parameters, such as heart rate variability as a representation of autonomic tone. In practice, no single hormone or physiological response is ideal for measuring psychogenic stress, and it has been suggested that multiple parameters should be used simultaneously to improve accuracy [10]. There are no known physiological measurements of stress which can serve as specific markers of distress; measuring stress is made difficult by confounding factors, such as measurements that describe the function of the immune system in addition to characterizing the stress response. Behavioral measures of stress suffer from similar non-specificity, and are in many ways less well understood than physiological measures.

### 5.1. Physiological Measures of Stress

Physiological measurements of stress include assessment of the HPA axis (usually cortisol measurement), salivary immunoglobulin (Ig)A (sIgA), and the neutrophil:lymphocyte (N:L) ratio. The benefits and limitations of each of these are discussed below. Additionally, Table 1 summarizes some physiological measures of stress in regards to their invasiveness, the time period that they reflect, and daily variation.

**Table 1:** Physiological measurements of stress in dogs.

Type	Invasiveness	Time period reflected	Daily variation
Plasma cortisol	Moderate	3–40 minutes	High
Salivary cortisol	Low	4–40 minutes	High
Urinary cortisol	None	6–12 hours	Low
Salivary IgA	Low	0–30 minutes	High
N:L ratio	Moderate	Hours	Low
HRV	Moderate	Hours	High

#### 5.1.1. The HPA Axis

Measurement of HPA axis activity through concentrations of GCs such as cortisol and corticosterone is the most widely used hormonal measurement of psychogenic stress, and is commonly used in humans and dogs [58,59,60]. Cortisol concentrations in the plasma, saliva, or urine have been shown to significantly increase in dogs 15–30 minutes after the onset of a stressor, indicating increased HPA axis activation [61]. Because blood collection is moderately invasive, many animals will display increased cortisol concentrations after immobilization and venipuncture. However, cortisol secretion in response to collection does not affect the values in dogs if venipuncture and collection can be completed in less than 3 minutes, or if saliva collection can be completed in less than 4 minutes [62]. Salivary cortisol concentrations are frequently used in stress studies of dogs because they are strongly correlated with plasma cortisol, are less invasive, and require less training of the handler performing the collection [59,63]. Urine contains both cortisol and its metabolites, and may be collected non-invasively. Urine cortisol:creatinine ratios provide a summary of HPA axis activity over several hours, and are therefore more useful for measurement of chronic stress than of HPA axis response to an acute stressor [64]. 

The evaluation of HPA axis activation by means of cortisol concentration measurement has multiple limitations. Measurement of cortisol activity reflects physical and/or psychological arousal, and therefore is not a specific measurement of psychogenic stress. The level of psychogenic distress experienced by an individual may be only moderately associated with cortisol concentrations, due to the intricate relationship of HPA axis function and metabolic demands [59,65]. In fact, activation of the HPA axis may indicate positive stress rather than distress, as it is activated in dogs after time in the dog park [66] or in sled dogs anticipating a race [67]. Moreover, due to the degree of variability in cortisol concentrations between individuals, no reliable species reference ranges exist, and basal concentrations must be evaluated with reference to a control group [68]. Daily variability in cortisol concentrations is such that sampling the same animal on multiple days is recommended where possible [62]. Additionally, samples should be obtained at the same time of day to avoid possible diurnal variability [69]. These samples may be affected by daily experience, including sleep patterns, meal content, and recent exercise [69,70,71].

To some extent, study design can mitigate the issue of variability in cortisol concentrations, by pairing similar subjects in statistical analysis, taking multiple samples, taking samples at the same time of day, and by limiting the variety of ages, breeds, and life experience where possible. However, cortisol alone is not a highly reliable measurement of stress. Despite its common use, it has proven to be a problematic tool at best, due to its extreme inter- and intra-individual variability.

#### 5.1.2. Salivary Immunoglobulin (Ig) A

Salivary immunoglobulin(Ig)A (sIgA) is a well-known marker of stress in humans [72]. sIgA concentrations have been shown to negatively correlate with cortisol concentrations in dogs, and have been used to evaluate both acute and chronic stress in dogs [73,74]. Limitations of using sIgA concentrations to assess stress are that they change rapidly, will decline immediately after the presentation of a stressor, have significant diurnal variation, and may fail to distinguish between physiologic and psychogenic stress [73,75]. Because sIgA concentrations significantly correlate with plasma cortisol concentrations, they may be subject to similar variation, though causes of variability of sIgA are not as well described in domesticated animals as are causes of cortisol variability [74].

#### 5.1.3. Neutrophil: Lymphocyte (N:L) Ratio

Because both acute and chronic stress are known to be associated with neutrophilia and lymphopenia, an increased neutrophil:lymphocyte (N:L) ratio has been used as a marker of stress in human and veterinary studies, including studies in dogs [8,76]. Using the N:L ratio has the advantage of a several-hour time delay from the onset of the stressor, so that it may be used to measure baseline stress levels in dogs who have recently arrived at a veterinary hospital. 

Limitations of the N:L are similar to those of GC concentrations, in that it cannot distinguish between physiologic and psychogenic stress and is invasive; therefore, this marker may not be appropriate for all studies [76]. This ratio suffers from less variability than cortisol concentrations, but is correspondingly less responsive in the short term to the effects of very acute stressors [76]. Additionally, its use in veterinary studies of stress is much less common and less well understood than the use of cortisol.

#### 5.1.4. Heart Rate and Heart Rate Variability

Sympathetic nervous system activation during psychogenic stress results in increased heart rate, which has been used to assess acute stress in dogs [3,77,78,79]. However, heart rate elevation has been found to be a non-specific marker of distress, increasing in cases of positive and negative stress, as well as with increased motor activity [3,77,79]. Heart rate ordinarily varies over time due to the prevailing balance of parasympathetic and sympathetic nervous system input. During stressful events, heart rate variability (HRV) declines as the heart rate remains mostly elevated, and this parameter can be measured and interpreted. Decreased HRV has been shown to be present in dogs which may be suffering from stress due to hospitalization [9]. Heart rate variability has also been used as a marker of acute stress in humans [80] and lab animals [24], and may prove valuable as a marker of acute stress in dogs. Heart rate variability measurement requires continuous monitoring, typically accomplished via attachment of telemetry equipment [78,79], but more simple and feasible methods developed for human athletes hold promise for studies of stress in dogs [81,82]. Ideally, dogs should be acclimated to wearing telemetry devices, in order to avoid the confounding of the response to the experimental stressor with a reaction to the equipment [77].

#### 5.1.5. Additional Physiologic Measures

ACTH, norepinephrine, and epinephrine [8,83] have been used as biomarkers of stress in dogs, but only rarely. Additional measures of physiologic change have been documented in dogs post-surgically, including decreased prolactin, monocytosis, eosinopenia, and increased C-reactive protein and haptoglobin [23], but these measures have not been used as biomarkers of stress in dogs.

#### 5.1.6. Limitations of Physiologic Measurements of Stress

An understanding of the limitations of measurement of physiologic changes to evaluate psychogenic stress is important when designing stress studies using these measurements. What one individual experiences as a stressor, another may not, and stimuli which evoke a stress response in one individual may not do so in another. A standardized stress stimulation tool in humans, the Trier Social Stress Test, produces a stress response measurable with cortisol in only 70–80% of tested individuals [84]. Similarly, a hospital environment triggering a stress response in one dog may not do so in another individual coming from a comparatively more stimulating or stressful home environment. In other words, though physiologic changes are likely to reflect an animal’s distress, prediction of what constitutes a stressor for a specific individual may be difficult if the animal’s prior history is unknown. Conclusions from studies using purpose-bred dogs of uniform age, breed, gender, and life experience should be applied to pet dogs, a population which might vary in all of those parameters, only with caution. On the other hand, studies on pet dogs may be confounded by variability in such a large number of parameters that the necessary sample size to reach statistical significance may be much larger than for uniform populations.

### 5.2. Behavioral Markers of Stress in Dogs

Behavioral measures of stress have been used to inform interpretation of physiological markers of stress such as cortisol concentrations or heart rate, and are non-invasive [4,58,60,64]. However, behavior may be difficult to interpret, and may not be constant for different stressors [85]. In one study of stress in dogs moved to a new kennel environment, behaviors thought to be associated with stress showed no correlation with urinary cortisol concentrations [60]. The interpretation of individual behaviors may be contextual, so that behaviors may be linked to stress in some situations but not others. Oral activity such as lip licking increases after some acute stressors, including pulling the dog’s head down by a rope, opening an umbrella, or pressing the dog down to the floor [86]. However, oral activity does not increase after other acute stressors, such as a loud sound or the presentation of a moving toy car [60]. Similarly, paw lifting is increased in dogs subjected to austere housing compared to dogs with more enriched housing, but not in dogs introduced to an animal shelter [60,85]. Therefore, stress behaviors cannot be grouped into categories such as “behaviors in response to acute stress” or “behaviors in response to housing stress,” but must be considered for a particular environment.

### 5.3. The Need for Behavioral Stress Assessment Instruments for Hospitalized Dogs

Hospitalization represents a predictable set of stressors, including separation from the caretaker and exposure to novel surroundings, which may have an impact on the outcome of medical treatment. If behaviors were determined to correlate positively or negatively with cortisol concentrations or other physiologic markers, generation of stress assessment scales or instruments may be possible. Validation of scales is necessary, to guard against errors in the development process, and to ensure reproducibility.

Validated behavioral instruments have been used to assess and study other states in dogs, such as acute pain, postoperative pain, and pain from osteoarthritis [86,87,88]. Pain is a state that, like stress, has no “gold standard” for detection in non-verbal beings, even experimentally, but the emergence of pain scales in veterinary medicine has enhanced the study of interventions and assessments greatly in recent years. 

Published studies of stress in dogs commonly involve measurement of salivary cortisol. Due to cortisol’s variability and non-specificity as a marker of stress, the addition of a second marker may increase the reliability of behavioral instruments to detect stress [10]. However, the measurement of multiple physiologic variables represents a challenge in the clinical study of patient populations, particularly when such measures may themselves perturb the state of the system being studied in the subject. 

In addition to allowing the study of the effects of stress on health care outcomes, behavioral assessment may provide a useful clinical diagnostic measure. Salivary cortisol is not useful for “point of care” detection of stress levels in hospitalized patients, since measurement requires an immunoassay and strict controls. Clinical evaluation of stress levels of patients currently involves interpretation of behavior [23]. Our recent work has contributed to the literature by providing some evidence for correlation of specific behavior combinations associated with salivary cortisol in hospitalized dogs [89]; however, it was necessary to observe the dogs over a period of time (20 minutes), and this was considered too cumbersome for a useful clinical observation period. Behavior is an inexpensive and non-invasive parameter to assess, but due to its high variability and lack of understanding of the specific sources of that variability in different situations, it may be difficult to interpret [85]. Therefore, in order for behavior to be a useful indicator, there is a need for more investigation into correlations between stress markers and behavior in hospitalized dogs.

## 6. Conclusions

Both because of a growing recognition of its potential to affect health outcomes in humans and animals, and because the experience of psychogenic distress is unpleasant, veterinarians should recognize the importance of identification and reduction of psychogenic distress in their patients. Therefore, there is a need for investigation into methods of measurement of stress in hospitalized animals. If we can produce behavioral assessment instruments, or “stress scales,” to enable identification of highly stressed patients, then this may aid in determining the extent to which negative outcomes are associated with increased stress and methods to reduce its impact on the patient. As evidence accumulates in support of the fact that psychogenic stress has important health consequences across a range of species, a general understanding on the part of clinicians, as well as investigation of its recognition and means of mitigation using validated stress assessment tools, are important features of our evolving quest to improve the well-being and health of veterinary patients.
